# Richmond (Va.) Medical Journal

**Published:** 1868-05-01

**Authors:** 


### *Richmond (Va.) Medical Journal.*

This excellent monthly, which failed to reach our *sanctum* for so long a period that we feared its suspension, comes again to hand in excellent style, and with varied and valuable contents. It is edited by E. S. Gaillard, M.D., Professor in the Medical College of Virginia. It is now the largest medical monthly in America. Liberal club and premium rates are offered. We will furnish subscribers with *both* the *Chicago* and *Richmond Medical Journal*, at $5.00 per annum. Address the Editor of this journal, or Professor E. S. Gaillard, M.D., locked box 32, Richmond, Va.

☞ Will Dr. Gaillard address his exchange to this journal, Box 1948? We lose many of our most valued exchanges by carelessness in distribution at the P.O., meanwhile receiving and being obliged to return a parcel of homœopathic and other trash, which interests us in no wise.

